# A statistical algorithm showing coenzyme Q_10_ and citrate synthase as biomarkers for mitochondrial respiratory chain enzyme activities

**DOI:** 10.1038/s41598-016-0008-1

**Published:** 2016-12-05

**Authors:** D. Yubero, A. Adin, R. Montero, C. Jou, C. Jiménez-Mallebrera, A. García-Cazorla, A. Nascimento, M. M. O’Callaghan, J. Montoya, L. Gort, P. Navas, A. Ribes, M. D. Ugarte, R. Artuch

**Affiliations:** 1 Institut de Recerca Pediàtrica-Hospital Sant Joan de Déu (IRP-HSJD), Barcelona, Spain; 2 Departamento de Estadística e I.O., Universidad Pública de Navarra, Pamplona, Navarre Spain; 3 Institute for Advanced Materials (InaMat), Universidad Pública de Navarra, Pamplona, Navarre Spain; 4 Centro de Investigación Biomédica en Red (CIBERER), ISCIII, Barcelona, Spain; 5 Departamento de Bioquímica, Biología Celular y Molecular. Universidad de Zaragoza, Zaragoza, Spain; 6 Institut de Bioquímica Clínica, Corporació Sanitària Clinic, Barcelona, Spain; 7 Centro Andaluz de Biología del Desarrollo, Universidad Pablo de Olavide, Sevilla, Spain

## Abstract

Laboratory data interpretation for the assessment of complex biological systems remains a great challenge, as occurs in mitochondrial function research studies. The classical biochemical data interpretation of patients versus reference values may be insufficient, and in fact the current classifications of mitochondrial patients are still done on basis of probability criteria. We have developed and applied a mathematic agglomerative algorithm to search for correlations among the different biochemical variables of the mitochondrial respiratory chain in order to identify populations displaying correlation coefficients >0.95. We demonstrated that coenzyme Q_10_ may be a better biomarker of mitochondrial respiratory chain enzyme activities than the citrate synthase activity. Furthermore, the application of this algorithm may be useful to re-classify mitochondrial patients or to explore associations among other biochemical variables from different biological systems.

## Introduction

Mitochondrial disease diagnosis is a complex process that relies on clinical, biochemical, neuroimaging, histological, and molecular data. Skeletal muscle is considered the most suitable tissue for the diagnosis of these disorders because of its availability and high metabolic rate. Biochemical measures of mitochondrial respiratory chain (MRC) enzyme activities are crucial for clinical diagnosis. The activities of MRC complexes (I–IV) are assayed spectrophotometrically, and the results are normalized to the total muscle protein content or to the activity of the mitochondrial matrix enzyme citrate synthase (CS), which is commonly used as an index of mitochondrial abundance^1^. Normalization to CS activity will facilitate the detection of partial enzymatic defects in diseases with compensatory mitochondrial proliferation^2^, while normalization to total proteins could unmask an MRC enzymatic defect in some cases of mitochondrial DNA depletion syndromes that may be associated with low-normal CS activities^3–6^.

Coenzyme Q_10_ (CoQ) is a lipid with a key role in mitochondrial oxidative phosphorylation because it is essential for electron transport from complex I and II to complex III of the MRC. The link between CoQ deficiency and dysfunction of complexes I + III and II + III is expected because low CoQ availability in mitochondria would impair the electron transfer essential for ATP production^7–10^. Muscle CoQ concentrations have been demonstrated to be associated with CS activity^10, 11^, as well as with other mitochondrial dysfunction biomarkers, such as the percentage of subsarcolemmal mitochondrion aggregates^10^. Furthermore, it has been suggested that total muscle CoQ is the best predictor of an MRC abnormality^12^. These findings strongly support the hypothesis that routine quantitative evaluation of muscle CoQ might be a new tool for both estimating MRC enzyme activities in muscle biopsies and diagnosing CoQ deficiency states^10, 12^.

With this background, our aim was to develop and apply an exploratory statistical procedure to assess muscle CoQ content and CS activity as biomarkers of mitochondrial activity evaluated by the analysis of MRC enzyme activities. After the initial statistical assessment, subpopulations of individuals displaying a high linear correlation coefficient among the different biochemical variables were identified.

## Material and Methods

### Patients

During the last 15 years, we have studied 448 muscular biopsies from patients suspected of mitochondrial disorders (age range 1 month-16 years; mean: 3.6 years). Results of both CoQ and CS were available in 447 samples. Of this population, 179 showed normal results for CoQ levels and all MRC enzymes and citrate synthase activities. Data were compared with those of a previously reported control population (N = 37; age range 2–16 years; average 9.2 years)^11^.

### Ethical issues

The study was approved by the ethical committee of Hospital Sant Joan de Déu. Patients or their parents signed informed consent. All methods, including the obtaining of tissue samples from patients and controls, were carried out according to the Helsinki Declaration of 1964, as revised in 2001.

### Biochemical studies

Muscle biopsies were taken and prepared according to standard procedures. NADH:cytochrome c oxidoreductase (complex I + III), succinate:cytochrome c reductase (complex II + III), succinate dehydrogenase (complex II), ubiquinol-cytochrme C oxidoreductase (complex III), cytochrome C oxidase (complex IV) and CS activities were determined using described spectrophotometric methods^13, 14^. Enzyme activity results were expressed as nmol/min* mg of protein and mUnits/CS Units. Total muscle CoQ levels were determined by reverse-phase high-pressure liquid chromatography (HPLC, Waters, MA, USA) with electrochemical detection (Coulochem II, ESA, MA, USA) (Montero *et al.*, 2008). The CoQ values were expressed as nmol/gram of total protein content measured by the Lowry method^15^.

### Statistical methods

Pearson linear correlation coefficients were initially computed between MRC enzyme activities, CS activity and CoQ content in muscle homogenates from patients. Statistical significance was evaluated using p < 0.01. Calculations were performed using the R program (version 3.2.3). See, for example Ugarte *et al.* ^16^.

Two statistical procedures were developed to further explore the correlations detected amongst the different biochemical variables. In particular, two algorithms were implemented to identify subpopulations of individuals in which a high correlation was reached (r > 0.95).

1. Agglomerative procedure: Initial linear axes were found using the robust algorithm developed by García-Escudero *et al.* ^17^, which was specifically designed to detect linear clusters. Then, an iterative procedure was implemented by adding individuals to the initial axes until a fixed high correlation was achieved. The procedure was specifically constructed as follows.
  Let *X* and *Y* be the variables of interest (that initially showed linear association), and let *M* ^(0)^ be the set consisting of the three nearest points to the simple regression line Y_i_ = *β*_0_ + *β*_1_X_1_ + *ε*_i_ (calculated by ordinary least squares) fitted over the individuals selected by the linear clustering method implemented by García-Escudero *et al.* ^17^; i.e., the three individuals with the smallest residuals $|\hat{\varepsilon }|=|{Y}_{i}-{\hat{Y}}_{i}|$ are selected. Next, a “correlation loss measure” between the set *M* ^(0)^ and any other individual *i* is defined as
  1 $$CLM({M}^{(0)},i)=\rho ({X}_{{M}^{(0)}},{Y}_{{M}^{(0)}})-\rho ({X}_{{M}^{(0)}\cup \{i\}},{Y}_{{M}^{(0)}\cup \{i\}}),$$
  where $\rho ({X}_{{M}^{(0)}},{Y}_{{M}^{(0)}})$ is the Pearson’s correlation coefficient between the variables *X* and *Y* within the set *M* ^(0)^.
  1. Let ${M}^{(1)}={M}^{(0)}\cup \{{i}^{\ast }\}$ be a new set where *i* ^*^ is the individual that verifies
  2 $$CLM({M}^{(0)},i\ast )\le CLM({M}^{(0)},i),\,\,{\rm{for}}\,{\rm{all}}\,i$$
  i.e., *i* ^*^ is the individual with the smallest correlation loss.
  2. Step 1 is repeated while $\rho ({X}_{{M}^{(k)}}{,}_{{M}^{(k)}})\ge {r}^{\ast }$, where *r* ^*^ is the desired correlation to be reached.
2. Divisive procedure: Unlike the agglomerative procedure, there is no need to define an initial axis because all of the individuals are considered as starting points in this method. Then, an iterative procedure is implemented by deleting one case (individual) at each step until a fixed high correlation is achieved.

More explicitly, the procedure can be described as follows:

Let *M* ^(0)^ be the set of all individuals in our target population. We define the “correlation gain measure” between the set *M* ^(0)^ and the individual *i* as,

3 $$CGM({M}^{(0)},i)=\rho ({X}_{{M}^{(0)}-\{i\}},{Y}_{{M}^{(0)}-\{i\}})-\rho ({X}_{{M}^{(0)}},{Y}_{{M}^{(0)}})$$

where $\rho ({X}_{{M}^{(0)}-\{i\}},{Y}_{{M}^{(0)}-\{i\}})$ is the Pearson’s correlation coefficient between the variables *X* and *Y* within the set *M* ^(0)^ without the *i* individual.

1. Let *M* ^(1)^ = *M* ^(0)^ − {*i* ^*^} be a new set where where *i* ^*^ is the individual that verifies

4 $$CGM()({M}^{(0)},{i}^{\ast })\ge CGM({M}^{(0)},i),\,{\rm{for}}\,{\rm{all}}\,i$$

that is, the individual with the largest correlation gain.

2.Step 1 is repeated k times until $\rho ({X}_{{M}^{(k)}},{Y}_{{M}^{(k)}})\ge {r}^{\ast }$, where *r* ^*^ is the desired correlation to be reached.

Both algorithms were implemented in R (version 3.2.3). After the application of the two algorithms, subpopulations displaying high correlations were identified.

## Results

Biochemical results in muscle biopsies in the entire cohort of patients together with our reference values are stated in Table 1. Primary data about CoQ values, CS and CIII activities are stated in Supplementary Table 1.

**Table 1:** Biochemical data from the whole cohort of patients.

	N	Range (SD)	Mean	Reference values
CoQ	447	14.8–1253 (114)	185	110–480 nmol/gr protein
CI + III	429	1–425 (23)	19	12–56 nmol/min*mg protein
CII + III	434	0.5–142 (9)	9	7–24 nmol/min*mg protein
CII	339	1–86 (6)	6	4–10 nmol/min*mg protein
CIII	305	0.1–1180 (77)	75	55–259 nmol/min*mg protein
CIV	299	4–215 (40)	82	59–170 nmol/min*mg protein
CS	448	12.9–522 (63)	132	71–200 nmol/min*mg protein
CoQ/CS	436	0.1–49 (3)	4	2.68–8.40 nmol/CS Units
CI + III/CS	443	0.1–826 (109)	160	107–560 mUnits/CS Units
CII + III/CS	447	3.0–877 (60)	76	60–149 mUnits/CS Units
CII/CS	265	7.0–757 (48)	46	33–69 mUnits/CS Units
CIII/CS	240	0.1–1720 (290)	511	498–1760 mUnits/CS Units
CIV/CS	310	100–1872 (301)	669	503–1300 mUnits/CS Units

The range, standard deviation (SD) and mean of CoQ and MRC activities is given, expressed either by protein content or CS units. Reference values are also stated.

Pearson correlation coefficients and significance values of primary data are stated in Tables 2 and 3 (data were normalized to muscle total protein concentration, CoQ values and CS activity). CoQ correlation coefficients with all the MRC enzyme activities were higher compared with those obtained between CS and the other MRC enzymes (Table 2). When the CoQ concentrations and MRC enzyme activities were normalized to CS activity values, the highest correlation coefficient was observed between CoQ and complex II + III (Table 3). Then, in the whole population, we used CoQ as a normalizer of the CS and MRC activities. We saw that the MRC complexes that correlate more with CS activity were II, III and IV (Table 3).

**Table 2:** Correlation data (Pearson test) among muscle CoQ (nmol/g protein), CS (nmol/min*mg protein) and MRC activities (nmol/min*mg protein) from the whole population.

	CoQ	CS
CoQ	—	r = 0.457 (N = 447); p < 0.0001
CS	r = 0.457 (N = 447); p < 0.0001	—
CI + III	r = 0.202 (N = 428); p < 0.0001	r = 0.055 (N = 429); p = 0.259
CII + III	r = 0.288 (N = 433); p < 0.0001	r = 0.113 (N = 434); p = 0.019
CII	r = 0.316 (N = 338); p < 0.0001	r = 0.238 (N = 339); p < 0.0001
CIII	r = 0.228 (N = 304); p < 0.0001	r = 0.137 (N = 305); p = 0.017
CIV	r = 0.466 (N = 298); p < 0.0001	r = 0.423 (N = 299); p < 0.0001

**Table 3:** Correlation data (Pearson test) among muscle CoQ, CS and MRC activities normalized to either CS activity (middle column) or CoQ content (right column).

	CoQ/CS	CS/CoQ
CI + III/CS	r = 0.479 (N = 433); p < 0.0001	—
CI + III/CoQ	—	r = 0.236 (N = 428); p < 0.0001
CII + III/CS	r = 0.686 (N = 435); p < 0.0001	—
CII + III/CoQ	—	r = 0.254 (N = 433); p < 0.0001
CII/CS	r = 0.179 (N = 265); p = 0.003	—
CII/CoQ	—	r = 0.345 (N = 338); p < 0.0001
CIII/CS	r = 0.193 (N = 240); p = 0.003	—
CIII/CoQ	—	r = 0.460 (N = 304); p < 0.0001
CIV/CS	r = 0.276 (N = 310); p < 0.0001	—
CIV/CoQ	—	r = 0.555 (N = 298); p < 0.0001

### Agglomerative and divisive algorithms

**Figure 1:**
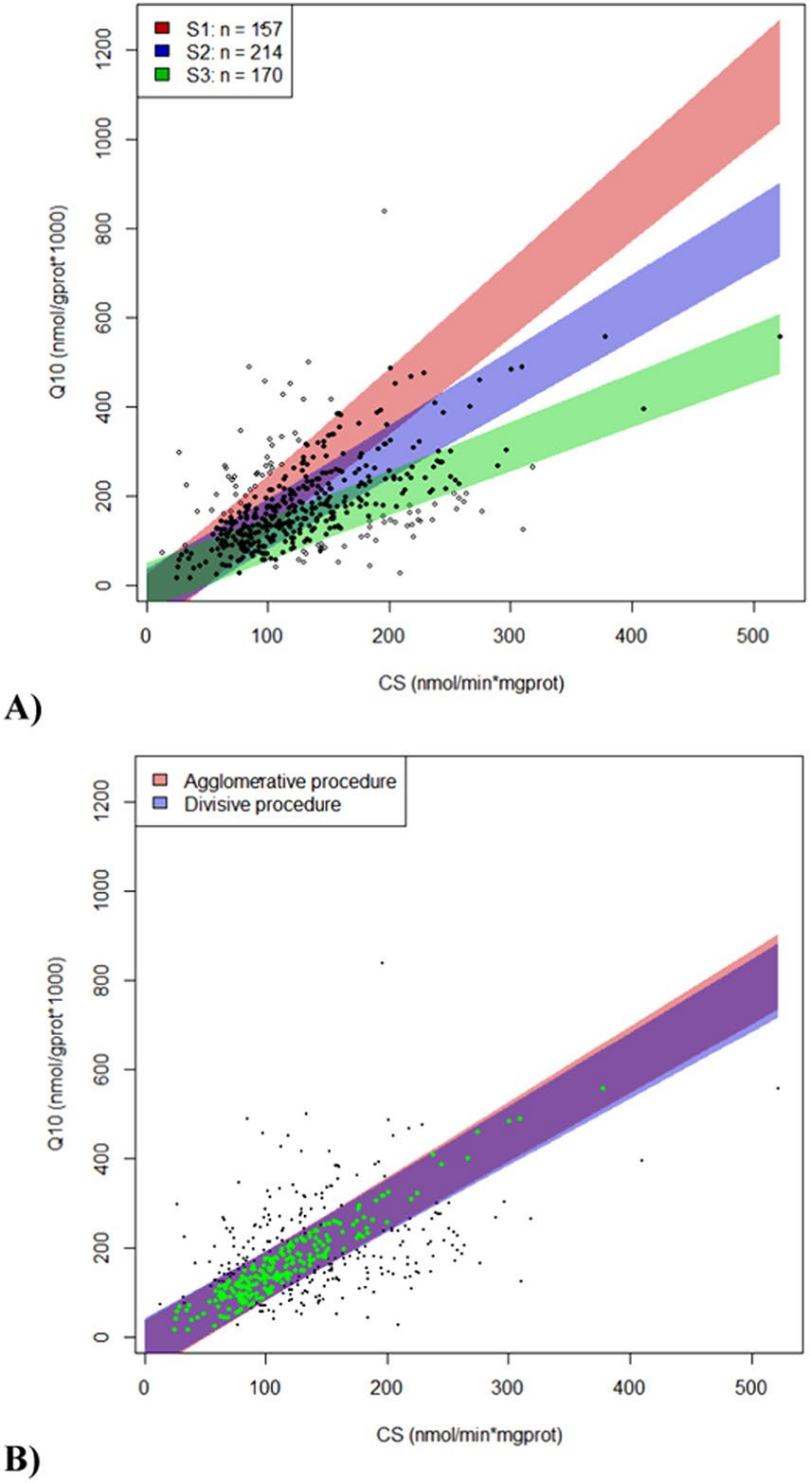
Statistical study between muscle CS activity and CoQ values. (**A**) Three possible clusters identified by the agglomerative method. The cluster selected was the one with the highest number of individuals (blue). (**B**) Good agreement was observed between agglomerative and divisive methods.

From the whole population (n = 447), observations with missing data and potential outliers were removed (ranging from 1 to 7 values depending on the variable). When applying the agglomerative procedure, three initial linear axes were used to find the corresponding clusters of individuals with a high correlation (r > 0.95) between the variables CoQ and CS. The cluster with the highest number of individuals was chosen. The divisive method identified essentially the same subpopulation (98% of individuals in common, n = 214) (Fig. 1).

We started with MRC enzyme activities and their association with either CS or CoQ. In the agglomerative method, three initial linear axes were considered, except for CS and complexes I + III and II + III, in which the agglomerative algorithm did not provide any sensible results and only the divisive method was applied. We finally selected the population with the highest number of individuals and compared it with those cases selected by the divisive method, which offered only one solution. Most MRC enzyme data showed that, when correlated with CoQ, populations with a higher number of individuals were detected (Table 4), especially for CoQ-dependent enzymes (CI + III and CII + III). The percentage of cases sharing the same correlation (r > 0.95) using agglomerative and divisive methods was also higher for CoQ when compared with CS, except for complex III (Table 4).

**Table 4:** Number of patients identified as having a correlation coefficient >0.95 among the different MRC enzyme activities vs CoQ or CS, and the degree of agreement between the agglomerative and divisive methods in the number of calculated individuals, except for CS, Complexes I + III and II + III, in which the agglomerative algorithm did not provide any sensible results and only the divisive method was applied.

MRC enzyme activities	CoQ (n and %)	CS (n and %)
CI + III	150 (85%)	129 (−%)
CII + III	180 (93%)	140 (−%)
CII	169 (98%)	149 (97%)
CIII	123 (100%)	129 (100%)
CIV	139 (86%)	128 (85%)

## Discussion

This is the first report to analyze CoQ and other MRC biomarkers in a large cohort of samples. We have developed a statistical algorithm to assess the feasibility of using both CoQ and CS as biomarkers for MRC activities.

After applying the first statistical approach to the different biochemical variables (Pearson single correlation test) across the whole cohort of patients, several observations were made: 1) Correlation of CoQ values with CS was high. 2) Correlation of CoQ values and MRC was strongest than correlation between CS activity and MRC (data normalized to total protein content). 3) By using other normalization strategies for MRC (with either CS or CoQ values), as expected, CoQ was highly correlated with CII + III when these activities were normalized with CS activity, and CS showed a high correlation coefficient with both CIII and IV activities when they were normalized to CoQ content. Thus, differences between the association of CoQ and CS with the different MRC activities were evident.

After these preliminary observations, the next step was to develop the algorithms to further explore these associations. We did not consider age as a potential confounding variable because it has been suggested that it is not related to the activities of most MRC enzymes and CS activities^18^.

The initial step of the agglomerative method looked for robust linear clusters to determine initial linear directions to start the iterative steps. Although it only provided a single linear cluster as the optimal solution, we decided to explore three different linear cluster solutions and applied the iterative steps to these three potential solutions. The cluster with the highest number of individuals was chosen. To further validate the agglomerative algorithm, we compared the final results with a divisive method, which offered a unique solution from the whole cohort of patients. Interestingly, the degree of agreement between the solutions provided by the two algorithms was very high (see Table 3). The algorithm was then able to provide subpopulations of individuals with a high linear correlation coefficient between the variables of interest. In our case, a single subpopulation seemed to be the most reasonable solution.

We chose a correlation coefficient value of 0.95 because it is remarkably high when we consider biological variables of this complexity. Notably, the number of cases where this correlation was detected was high, especially for CoQ and CS correlation, supporting the hypothesis that CoQ may be employed as a marker for MRC activity normalization. Therefore, the normalization of MRC activities to CoQ seems advisable for a better classification of mitochondrial patients and it would be a good predictor of MRC alterations^10^.

From the data shown in Table 4, we observed, in terms of number of individuals displaying a correlation >0.95, that CoQ was a better marker than CS for CI + II, CII + III and CII activities, while CS was better for CIII and similar for CIV activities. Furthermore, in all cases (except for CS, CI + III and II + III), the degree of agreement between agglomerative and divisive methods was very high, supporting the usefulness of this new statistical approach. One explanation for the fact that agglomerative algorithm did not provide any sensible results only for CS and complex I + III and II + III activities is probably because both MRC complexes need CoQ for a proper electron transfer. Moreover, the measurement of these complexes, especially that of CI + III, is technically complex.

Although a biological explanation is difficult, the proposed supramolecular organization of MRC could illustrate why CoQ may be a better mitochondrial biomarker than CS. The individual MRC complexes (except complex II) can assemble into different supercomplexes. Until now, the proposed supercomplexes consisted of the respirasome (complexes I, III, and IV), complexes I and III and complexes III and IV. Supercomplexes are presumed to be functional entities, which follow a fluidity model, and it is proposed to modulate respiratory chain efficiency and reactive oxygen species production^19^. By genetic modulation of interactions between complexes I and III and between complexes III and IV, it has been shown that these associations define a dedicated CoQ pool and organizes the electron flow to optimize the use of available substrates^20^. Additionally, defective mitochondrial enzymes that reduce CoQ such as Electron Transfer Flavoprotein Dehydrogenases (ETFDH) or depletion of mtDNA that induces defective respiratory complexes cause CoQ deficiencies^21^. It is then expected a high correlation between CoQ levels and respiratory complexes I, II and III.

In conclusion, we have developed a new algorithm for exploring associations among complex biological variables. In this specific example, we demonstrate that CoQ may be used as biomarker for MRC activities. Hence, its routine determination in the research of mitochondrial diseases seems advisable. Potential applications of the agglomerative method may be the re-classification of patients according to CoQ values, which might lead to a better understanding of mitochondrial disorders. Furthermore, this algorithm may be employed to identify populations displaying high correlations among other biological variables.

## Electronic supplementary material

Supplementary Table 1.
